# Short-Term Amoxicillin-Induced Perturbation of the Gut Microbiota Promotes Acute Intestinal Immune Regulation in Brown Norway Rats

**DOI:** 10.3389/fmicb.2020.00496

**Published:** 2020-03-26

**Authors:** Katrine Bækby Graversen, Martin Iain Bahl, Jeppe Madura Larsen, Anne-Sofie Ravn Ballegaard, Tine Rask Licht, Katrine Lindholm Bøgh

**Affiliations:** National Food Institute, Technical University of Denmark, Kongens Lyngby, Denmark

**Keywords:** antibiotics, microbiome, host response, Treg, regulatory T cells, IgA, goblet cells, mucus

## Abstract

The intestinal gut microbiota is essential for maintaining host health. Concerns have been raised about the possible connection between antibiotic use, causing microbiota disturbances, and the increase in allergic and autoimmune diseases observed during the last decades. To elucidate the putative connection between antibiotic use and immune regulation, we have assessed the effects of the antibiotic amoxicillin on immune regulation, protein uptake, and bacterial community structure in a Brown Norway rat model. Daily intra-gastric administration of amoxicillin resulted in an immediate and dramatic shift in fecal microbiota, characterized by a reduction of within sample (α) diversity, reduced variation between animals (β diversity), increased relative abundance of Bacteroidetes and Gammaproteobacteria, with concurrent reduction of Firmicutes, compared to a water control group. In the small intestine, amoxicillin also affected microbiota composition significantly, but in a different way than observed in feces. The small intestine of control animals was vastly dominated by *Lactobacillus*, but this genus was much less abundant in the amoxicillin group. Instead, multiple different genera expanded after amoxicillin administration, with high variation between individual animals, thus the small intestinal α and β diversity were higher in the amoxicillin group compared to controls. After 1 week of daily amoxicillin administration, total fecal IgA level, relative abundance of small intestinal regulatory T cells and goblet cell numbers were higher in the amoxicillin group compared to controls. Several bacterial genera, including *Escherichia/Shigella*, *Klebsiella* (Gammaproteobacteria), and *Bifidobacterium*, for which the relative abundance was higher in the small intestine in the amoxicillin group than in controls, were positively correlated with the fraction of small intestinal regulatory T cells. Despite of epidemiologic studies showing an association between early life antibiotic consumption and later prevalence of inflammatory bowel diseases and food allergies, our findings surprisingly indicated that amoxicillin-induced perturbation of the gut microbiota promotes acute immune regulation. We speculate that the observed increase in relative abundance of small intestinal regulatory T cells is partly mediated by immunomodulatory lipopolysaccharides derived from outgrowth of Gammaproteobacteria.

## Introduction

The gut associated lymphoid tissue is continuously exposed to a vast amount of bacterial and food derived antigens. Failure to develop tolerance toward these antigens may lead to inflammatory bowel diseases (IBD) or food allergies, both characterized by an adverse immune response directed against either commensal bacteria or dietary components. A healthy gut microbiota is important to avoid detrimental responses against harmless antigens (Stephen-Victor and Chatila, 2019), and epidemiological studies suggest that perturbation of the early life gut microbiota by pre- or post-natal exposure to antibiotics increases the risk of food allergies (Metsälä et al., 2013; Hirsch et al., 2017; Mitre et al., 2018) and autoimmune disorders including IBD (Hviid et al., 2011; Shaw et al., 2011; Kronman et al., 2012).

The gut microbiota promotes immune tolerance by stimulating the generation of regulatory T cells (Tregs) (Atarashi et al., 2011, 2013; Furusawa et al., 2013) and by reinforcing the intestinal barrier function, e.g., through regulation of tight junction permeability (Peng et al., 2009). Additionally, intestinal bacteria influence the secretion of mucus by goblet cells (Jung et al., 2015) and of immunoglobulin A (IgA) by plasma cells (Moreau et al., 1978; Shroff et al., 1995), both of which contribute to (I) antigen presentation and tolerogenic immune priming (Weltzin et al., 1989; McDole et al., 2012), (II) intestinal barrier function, and (III) balancing the microbial community (Martens et al., 2009; Donaldson et al., 2018). While multiple studies have assessed the influence of gut microbiota perturbation on immune regulation using broad-spectrum antibiotic cocktails (Ochoa-Repáraz et al., 2009; Hill et al., 2010; Nakamura et al., 2016) and germ-free (GF) animals (Ishikawa et al., 2008; Cahenzli et al., 2013), only few studies have investigated how administration of a single, clinically relevant antibiotic affects immune regulation (Tulstrup et al., 2015; Benakis et al., 2016).

Brown Norway (BN) rats constitute an well-established animal model in food allergy research (Abril-Gil et al., 2015; Jensen et al., 2019), as they mount an immune response resembling that of atopic human individuals (Knippels et al., 1999). The used BN rats originate from our in-house breeding colony, and in contrast to most animals from commercial vendors they have a natural microbiota and may thereby better resemble the human immune response (Rosshart et al., 2019). Amoxicillin is a bactericidal β-lactam antibiotic that affects both Gram-positive and Gram-negative bacteria by inhibiting an essential enzyme for bacterial cell wall synthesis (Bodey and Nance, 1972). It is the most widely used penicillin in Europe (European Centre for Disease Prevention and Control, 2018), and is frequently prescribed for treatment of pediatric infections (Dolk et al., 2018), and the impact of microbiota alterations caused by this antibiotic on early development of the immune system is therefore highly relevant to address. The gut microbiota of BN rats has not previously been analyzed by NGS based methods. In order to elucidate the interplay between microbiota composition and immune regulation, we characterized the temporal and spatial effects of 7 days of amoxicillin administration in BN rats by analyzing the gut microbiota, host immune regulation and intestinal permeability to cow’s milk protein. Interestingly, the observed rapid amoxicillin-driven perturbation of the gut microbiota was associated with an acute immune regulatory response.

## Materials and Methods

### Animals

BN rats from the in-house breeding colony (National Food Institute, Technical University of Denmark, Denmark) were housed in Makrolon cages and kept at a 12 h light:dark cycle, at a temperature of 22 ± 1°C and a relative humidity of 55 ± 5%. Rats were observed twice daily and clinical signs recorded. The rats were fed a milk-free diet for ≥10 generation that was produced in-house and based on rice flour, potato protein and fish meal as protein sources, as previously described (Bøgh et al., 2009), with the exception of maize flakes being substituted with rice flour. Diet and water were given *ad libitum*. Ethical approval was given by the Danish Animal Experiments Inspectorate and the authorization number given 2015-15-0201-00553-C1. The experiment was overseen by the National Food Institute’s in-house Animal Welfare Committee for animal care and use.

### Animal Experiment

BN rats of both sex were allocated into two groups of 12 rats. Starting from 4–5 weeks of age, rats were daily gavaged with 30 mg amoxicillin in 0.5 mL milli Q water or water alone from days 0 to 6. Fecal samples were collected 3 days before first gavage (day −3) and daily throughout the study and stored at −80°C for microbiota analysis. From days −3 and 7 one additional fecal sample was collected and stored on ice for total IgA quantification in fecal water. Intestinal uptake of β-lactoglobulin (BLG) from cow’s milk was assessed by dosing animals with 100 mg of whey protein concentrate (kindly provided by Arla Foods Ingredients) in 1 mL milli Q water by oral gavage 15 min prior to sacrifice.

### Dissection

Animals were euthanized by exsanguination using carbon dioxide inhalation as anesthesia. Whole blood was collected in sodium-heparin coated tubes (BD Bioscience, Franklin Lakes, NJ, United States) that were mixed for 10 min at room temperature (RT) and then stored on ice until processing immediately after the dissection, and in non-coated tubes stored 1 h at RT for preparation of serum. The intestine was excised and divided into multiple fractions for different analyses: mesenteric lymph nodes (mLNs) were stored in phosphate buffered saline (PBS, 137 mmol/L NaCl, 2.7 mmol/L KCl, 10 mmol/L Na_2_HPO_4_, and 1.8 mmol/L KH_2_PO_4_, pH 7.2) on ice, and processed for flow cytometry immediately after the dissection. From the first 20 cm of the small intestine (7 cm distal from the stomach), content was snap frozen in liquid nitrogen and stored at −80°C for microbiota analysis. The tissue was rinsed with 0.9% (w/v) NaCl before Peyer’s patches (PPs) and epithelium was removed, and the tissue was snap frozen in liquid nitrogen and stored at −80°C until analysis of permeability. PPs from the rest of the small intestine were excised, washed in 0.9% (w/v) NaCl and stored in PBS on ice until processing immediately after the dissection. The next 10 cm of the small intestine (without PPs) was rinsed with 0.9% (w/v) NaCl and stored in wash buffer [PBS with 2% (v/v) heat-inactivated fetal calf serum (FCS) and 15 mM HEPES] on ice until processing immediately after the dissection. The next 1 cm piece was rinsed with 0.9% (w/v) NaCl and stored in RNAlater (Invitrogen, Carlsbad, CA, United States) at −20°C until analysis of gene expression. Finally, 1 cm pieces of the small intestine and of colon were fixed overnight in 4% (w/v) paraformaldehyde for histology. Cecum was weighted, and cecum content was snap frozen in liquid nitrogen and stored at −80°C for microbiota analysis.

### Bacterial DNA Extraction

DNA was extracted from up to 200 mg of feces and cecum content and up to 250 mg of small intestine content by DNeasy PowerLyzer PowerSoil Kit (Qiagen, Hilden, Germany) according to the manufacture’s protocol. Mechanical lysis of bacteria was conducted at 30 cycles/s twice for 5 min on bead beater MM300 (Retsch, VWR, Haan, Germany). DNA concentrations were measured by the Qubit ds DNA BR kit (Life Technologies, Carlsbad, CA, United States) and DNA was stored at −20°C.

### Bacterial Load

Bacterial load was estimated by quantification of 16S rRNA gene copies by qPCR as previously described (Tulstrup et al., 2015). Briefly, the V3-region of the 16S rRNA gene was amplified in triplicate for each sample, using universal primers HDA1 and HDA2 (Walter et al., 2000). Each qPCR reactions contained 5.5 μL LightCycler1 480 II SYBR Green I Master (Roche Applied Science, Penzberg, Germany), 0.2 μM of each primer and 0.2 μL of diluted template DNA in a total reaction volume of 11 μL. Reaction conditions were: Pre-incubation at 95°C for 5 min followed by 45 cycles of 95°C for 10 s, 60°C for 15 s and 72°C for 45 s. Lastly, a melting curve was generated (95°C for 5 s, 68°C for 1 min and increasing the temperature to 98°C with a rate of 0.11°C/s with continuous fluorescence detection). The qPCR was run in 384-well format on a LightCycler^®^ 480 II (Roche Applied Science) and analyzed using the LightCycler^®^ 480 software. Tenfold dilutions of a linearized (*Sph*I-digested) plasmid standard, construction by cloning the 199bp V3-region of the 16S rRNA gene of *Escherichia coli* (ATCC 25922) into the pCR14Blunt-TOPO vector (Invitrogen), was used for quantification of 16S rRNA genes.

### Amplicon Sequencing of the 16S rRNA Gene

The bacterial community composition was analyzed by sequencing of the V3-region of the 16S rRNA gene in the extracted DNA. Amplification of the V3-region and subsequent sequencing was performed using the Ion Torrent PGM platform (Life Technologies) as previously described (Christensen et al., 2014). In short, the V3-region of the 16S rRNA gene was amplified using a universal forward primer (PBU 5′-A- adapter-TCAG-barcode-CCTACGGGAGGCAGCAG-3′) with a unique 10–12 bp barcode for each sample (Ion Xpress barcode as suggested by the supplier, Life Technologies) and a universal reverse primer (PBR 5′-trP1-adapter-ATTACCGCGGCTGCTGG-3′). The PCR reactions were conducted with 4 μL HF-buffer, 0.4 μL dNTP (10 mM of each base), 1 μM forward primer, 1 μM reverse primer, 5 ng template DNA in 1 μL, and 0.2 μL Phusion High-Fidelity DNA polymerase (Thermo Fisher Scientific, Waltham, MA, United States) in a total reaction volume of 20 μL. Reaction conditions were as follows: Initial 98°C for 30 s followed by 24 (feces and cecum content) or 30 (small intestine content) cycles of 98°C for 15 s and 72°C for 30 s, finally, 72°C for 5 min before cooling to 4°C. PCR products were purified by HighPrep^TM^ PCR Clean-up System (Magbio, Gaithersburg, MD, United States) according to the manufacture’s protocol, and DNA concentrations were determined with Qubit HS assay. Finally, a library was constructed by mixing an equal amount of PCR products from each sample. Sequencing was performed on a 318-chip for Ion Torrent sequencing using the Ion OneTouch^TM^ 200 Template Kit v2 DL.

### Sequence Data Handling

Sequence data was obtained in FASTQ format and further processed using CLC bio genomic workbench (Qiagen) in order to de-multiplex samples and remove sequencing primers. Further quality trimming using default settings (remove low quality nucleotides pbase-calling error = 0.05, trim ambiguous nucleotides = 2) and filtering only reads with a final length between 125–180 bp were exported in FASTQ format. Further quality trimming was performed in Divisive Amplicon Denoising Algorithm 2 (DADA2, Version 1.10.1) with default settings as described elsewhere (Callahan et al., 2016). Finally, an amplicon sequencing variant (ASV) table was constructed which contains the counts of each sequence variant in each sample. All sequence reads were taxonomically classified using the Ribosomal Database Project Multi-classifier tool (Wang et al., 2007).

The ASV table was imported into the “Quantitative Insights Into Microbial Ecology” (Qiime) 2 pipeline (Version 2019.1), and α and β diversity metrics were calculated by the function “diversity core-metrics-phylogenetic” based on a rooted phylogenetic tree. For analysis of fecal samples only, samples were rarefied to 20,000 reads to eliminate bias from uneven sampling depth, and for analyses that also included small intestine, samples were rarefied to 10,000 reads.

### Preparation of Serum and Fecal Water

To prepare serum, blood samples were allowed to coagulate for 1 h at RT and subsequently overnight at 4°C. The following day the coagulated blood was removed and samples were centrifuged at 1,800 *g* for 20 min at 4°C. The supernatants were transferred to clean tubes and stored at −20°C until analysis.

To prepare fecal water, fecal pellets were mixed with 10 μL cold PBS with 0.05% (w/v) NaN_3_ (Sigma, St. Louis, MO, United States) per mg sample in a bead beater MM300 (Retsch) for 15 min at 30 cycles/s followed by centrifugation at 16,000 *g* at 4°C for 10 min. The supernatants were transferred to clean tubes and stored at −20°C until analysis.

### Sandwich ELISA for Detection of Total IgA

For detection of total IgA antibodies sandwich ELISA was performed. Plates (96 wells MaxiSorp, NUNC, Roskilde, Denmark) were coated with 100 μL/well of mouse anti-rat IgA (MCA191, Bio-Rad, Oxford, United Kingdom) diluted 1:2,000 in carbonate buffer (15 mm Na_2_CO_3_, 35 mm NaHCO_3_, pH 9.6) and incubated overnight at 4°C. To block unspecific binding 200 μL/well of 3% (w/v) egg white protein (E0500, Sigma) in PBS with 0.01% (w/v) Tween 20 (PBS-T) was added and plates were incubated at 37°C for 1 h. Next, 50 μL/well of twofold serial dilutions of serum or fecal water samples and positive and negative control samples diluted in PBS-T were added, and plates were incubated for 1 h at RT. For detection, plates were incubated with 100 μL/well of horseradish peroxidase-conjugated goat anti-rat IgA (STAR111P, Bio-Rad, United Kingdom) diluted 1:10,000 in PBS-T. Between each step, plates were washed five times in PBS-T. After the last wash, plates were additionally washed twice in running tap water. The reaction was visualized by adding 100 μL/well of 3,3′,5,5-tetramethylbenzidine (TMB)-one substrate (Kem-En-Tec, Taastrup, Denmark) for 12 min and stopped with 100 μL/well of 0.2 M H_2_SO_4_. Absorbance was measured at 450 nm with a reference wavelength of 630 nm using a microtiter reader (Gen5, BioTek Instruments, Winooski, VT, United States). Antibody titers were expressed as the log2 titer values and defined as the interpolated dilution of the given serum sample leading to the mean absorbance for the negative control +3 standard deviation.

### Single Cell Suspensions of Small Intestinal and Lymphoid Tissues

Small intestine lamina propria (LP) samples stored on ice were opened longitudinally and cut into 5 mm pieces. The pieces were washed five times in wash buffer and tissue was digested in RPMI medium (Sigma) with 10% (v/v) FCS (Sigma), 15 mM HEPES (Sigma), 100 units/mL penicillin (Sigma), 100 μg/mL streptomycin (Sigma), 250 μg/mL collagenase (Sigma), and 1 mg/mL dispase II (Sigma) for 45 min on a shaker at 37°C. Afterward, the tissue digest was filtered through a 70 μm cell strainer, which was regularly rinsed with FACS buffer [PBS with 2% (v/v) FCS and 0.05% (v/v) NaN_3_]. PPs and mLNs were minced through 70 μm cell strainers, which were regularly rinsed with FACS buffer. The cell suspensions were centrifuged at 400 *g* (LP and mLNs) or 600 *g* (PP), 4°C for 10 min. The supernatants were discarded, and the cells re-suspended in 1 mL FACS buffer, and cells were counted in NucleoCassettes by use of a NucleoCounter (Chemometec, Allerød, Denmark).

### Staining of Lymphocytes for Flow Cytometry

Approximate 10^6^ cells/well were plated in 96-well plates. Non-specific binding was prevented by incubation 5 min on ice with 50 μL blocking solution with 10% (v/v) rat serum and 5 μg/mL anti-CD32 (D34-485, BD Biosciences) in FACS buffer. Cells were surface stained by addition of 50 μL/well antibody cocktail containing 2 μg/mL of each of the following: anti-B220-PE (HIS24, BD Biosciences), anti-CD3-PerCp-Cy5 (eBioG4.18, Thermo Fisher Scientific), anti-CD4-PE-Cy7 (OX-35, BD Biosciences), anti-CD45-APC-Cy7 (OX-1, Thermo Fisher Scientific), anti-CD25-BV421 (OX-39, BD Biosciences) in FACS buffer, and incubated 20 min on ice. Cells were washed with 100 μL FACS buffer and plates were centrifuged at 400 *g*, 4°C for 5 min. Supernatants were discarded and pellets resuspended in 200 μL FACS buffer.

Whole blood samples were diluted two times in FACS buffer and 100 μL were stained with 50 μL of the above described antibody cocktail and incubated for 20 min on ice. Afterward, 1 mL VersaLyse (Beckman Coulter, Brea, CA, United States) was added to lyse the red blood cells. The samples were incubated in dark at RT for 10 min before 2 mL FACS buffer was added and samples were centrifuge at 350 *g*, 4°C for 6 min. Supernatants were discarded and pellets resuspended in 200 μL FACS buffer and transferred to 96-well plates.

For identification of the transcription factors Forkhead box P3 (FoxP3) and Helios, cells were subsequently stained by using the BD Transcription Factor buffer set (BD Biosciences) according to the manufacture’s protocol. After fixation and permeabilization, the cells were blocked as described above. Cells were stained with 50 μL of 5 μg/mL anti-FoxP3-FITC (JFK-16s, Thermo Fisher Scientific) and 2 μg/mL anti-Helios- Alexa Fluor 647 (22F6, BD Biosciences) in TF Perm/Wash Buffer from the BD Transcription Factor buffer set, and incubated for 40 min on ice. After two washes in TF Perm/Wash Buffer, the cells were resuspended in FACS buffer. The stained cells were stored at 4°C until data was acquired. Just before accusation, 10 μL CountBright counting beads (Thermo Fisher Scientific) was added to each well. Data was acquired on BD FACSCanto II system (BD Bioscience) and analyzed by FlowJo (Version 10.4.2, BD Bioscience).

### Intestinal Protein Uptake

Total proteins were extracted from small intestine tissue samples by mixing with 10 μL tris-lysis buffer [150 mM NaCl, 20 mM Tris, 1 mM EGTA, 1% (v/v) Triton X-100, 1 mM EDTA] with 2% (v/v) Halt protease inhibitor cocktail (Thermo Fisher Scientific) per mg tissue. One stainless steel bead (Qiagen) were added to each sample, and samples were homogenized in buffer by TissueLyser II (Qiagen) at 30 cycles/s for 2 min. Samples were incubated on ice for 20 min and mixed by vortexing every 5 min. Samples were centrifuged at 15,000 *g* for 20 min at 4°C and the supernatants were frozen at −80°C until analysis. Concentrations of the cow’s milk protein BLG were quantified in tissue extracts and serum samples by a commercial bovine BLG ELISA kit (Bethyl Laboratories, Montgomery, AL, United States) in 96 wells MaxiSorp plates (NUNC) according to the manufacture’s protocol with the exception that plates were coated overnight. The concentrations were determined in triplicates and calculated from the duplicated standard curves generated for each plate.

### Histology

After overnight fixation in 4% formalin (CellPath, Newtown, United Kingdom), sections of small intestine and colon were dehydrated in a graded ethanol series from 77 to 99% ethanol (VWR Chemicals, Radnor, PS, United States). Xylene (VWR Chemicals) was used as clearing agent to replace the ethanol before the tissues were embedded in paraffin (Hounisen, Skanderborg, Denmark). Histological sections of 2 μm were stained with Hematoxylin (Ampliqon, Odense, Denmark) and Eosin (Merck, Darmstadt, Germany) to identify eosinophils, and Periodic acid–Schiff (PAS) (Merck) to identify goblet cells. The slides were examined using a Leica DMR upright microscope (Leica Microsystems GmbH, Wetzlar, Germany) and the software ImagePro Plus 7.0 (Media Cybernetics, Rockville, MD, United States) was used for images and measurements. Villus length was measured from the villus tip to the crypt-villus junction and in colon crypt depth was measured, with three villi or crypts measured/counted per animal. Analyses of histological sections were performed blinded.

### Tissue RNA Extraction, cDNA Synthesis, and RT-qPCR

Approximately 20–40 mg small intestine tissue, stored in RNAlater, was homogenized using TissueLyser II (Qiagen) and total RNA was extracted with RNeasy Mini Kit (Qiagen) with on-column DNase digestion (RNase Free DNase Kit, Qiagen) according to manufacturer’s protocol. RNA quality and quantity were assessed using NanoDrop. cDNA was synthesized from 500 ng RNA with Omniscript RT Kit (Qiagen) in accordance to manufacture’s protocol and in addition of Random Primer Mix (BioNordika, Herlev, Denmark) and Anti-RNAse (Ambion, Life Technologies, Carlsbad, CA, United States). Quantitative RT-PCR (RT-qPCR) reactions were run in technical duplicates using a Quantstudio 7 Flex Real Time PCR system (Applied Biosystems, Thermo Fisher Scientific) in 11 μL reactions containing 3 μL diluted cDNA (1:20), TaqMan Fast Universal PCR Master Mix (2×) (Applied Biosystems), and TaqMan gene Expression Assay (Applied Biosystems). TaqMan gene assays used were *Cdh1* (Cadherin-1 Rn00580109_m1), *Ocln* (Occludin Rn00580064_m1), *Cldn2* (Claudin 2 Rn02063575_s1), *Tjp1* (Tight junction protein 1 (ZO-1) Rn02116071_s1), *TSLP* (Thymic stromal lymphopoietin Rn01761072_m1), *Il33* (Interleukin 33 Rn01759835_m1), and *Muc2* (Mucin 2 Rn01498206_m1). Reaction conditions were: An initial cycle at 95°C for 20 s followed by 45 two-step thermal cycles at 95°C for 1 s and at 60°C for 20 s. The relative gene expression was calculated using the 2^–ΔCT^ method using *B2m* (Beta-2-microglobulin Rn00560865_m1) and *Sdha* (Succinate dehydrogenase complex Rn00590475_m1) as normalization genes. Data was acquired with Quantstudio 7 Flex software (Applied Biosystems, Foster City, CA, United States).

### Statistics

Differences between β diversity of groups were assessed by applying ANalysis Of SImilarities ANOSIM (Clarke, 1993) to weighted and unweighted UniFrac distances, and within group dispersion was analyzed with PERMDISP (Anderson et al., 2006) both in Qiime 2. Differential abundant genera between the amoxicillin and control group were determined by ANalysis of COMposition of Microbiomes (ANCOM) (Mandal et al., 2015) in R. This was only applied to small intestine samples, because more than 25% of genera varied between the two groups in cecum and feces. Graphs and additionally statistically analyses were generated in Prism version 8.1.1 (GraphPad, San Diego, CA, United States). Difference between initial and later time points within the same group were analyzed by one-way ANOVA followed by Dunnett’s multiple comparisons test. Difference between the two groups at the same time point were analyzed by *t*-test (when data passed D’Agostino-Pearson normality test) or non-parametric Mann–Whitney test (when not normally distributed). Non-parametric Spearman correlations were calculated between all pairs of relative abundance of small intestine genera (present in minimum 10 animals) and selected host response parameters. When indicated, *p*-values were false discovery rate (FDR) corrected by a two-stage sharpened method (Benjamini et al., 2006).

## Results

### Amoxicillin Transiently Reduces Fecal Bacterial Load and Persistently Decreases Fecal Microbial Diversity

**FIGURE 1:**
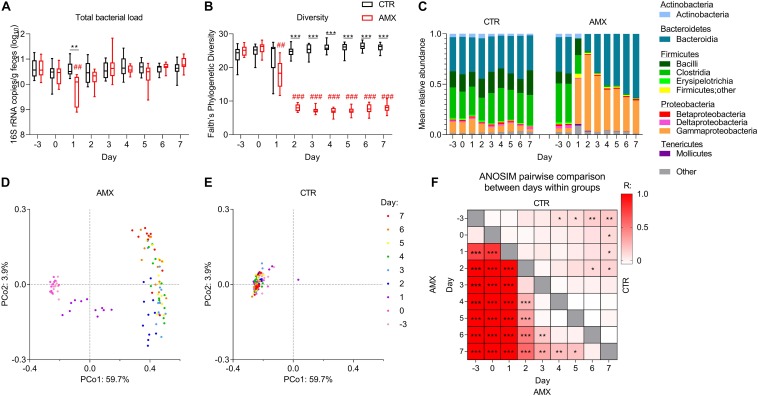
Temporal effects of amoxicillin on fecal bacterial load and composition. **(A,B)** Total bacterial load **(A)** and Faith’s phylogenetic diversity index **(B)** of control (CTR, black) and amoxicillin (AMX, red) groups. **(C)** Mean relative abundance of bacterial classes. **(D,E)** Principal coordinate analysis (PCoA) plots of unweighted UniFrac distances in the AMX **(D)** and CTR **(E)** groups colored according to sample day. **(F)** Heat-map of pairwise comparisons between days within the control group (upper right corner) and within the amoxicillin group (lower left corner) identified by Analysis of similarities (ANOSIM) between unweighted UniFrac distances. Intensity of the color indicates the value of the R statistics, where intense colors indicate large differences. Statistically significant differences between CTR and AMX on the same day analyzed by multiple *t*-test and FDR-correction **(A,B)** or between days analyzed by ANOSIM **(F)** are indicated as *p < 0.05, **p ≤ 0.01, ***p ≤ 0.001, and statistically significant differences relative to the initial level in the same group analyzed by one-way ANOVA followed by Dunnett’s multiple comparisons test **(A,B)** are indicated as ^##^*p* ≤ 0.01, ^###^*p* ≤ 0.001.

BN rats were gavaged daily with either amoxicillin or water for 1 week, and the temporal effects on fecal bacterial load and composition were followed. The 1st day after onset, a statistically significant reduction of total bacterial load was observed in the amoxicillin group as compared to both the baseline level (day −3) and to the level in the control group (Figure 1A). The effect of amoxicillin on bacterial load was transient since no differences were observed compared to the initial level nor to the control group from day 2 and onward. In order to assess the overall effect of amoxicillin on gut microbiota, the within sample (α) diversity was estimated by the Faith’s phylogenetic diversity index. A statistically significant reduction in diversity was observed in the amoxicillin group as compared to the initial level from day 1, as well as between the amoxicillin and control group from day 2 (Figure 1B).

### Amoxicillin Rapidly Disturbs Fecal Microbiota and Promotes Gammaproteobacteria Outgrowth

From day 1, the mean relative abundance of Firmicutes and Actinobacteria were lower in the amoxicillin group than in the control group, while Proteobacteria, especially Gammaproteobacteria were higher (Figure 1C and Supplementary Figures S1C, S2). In the amoxicillin group, the fecal microbiota continued changing over the course of the study, with the relative abundance of Bacteroidetes gradually expanding over time. This continuous shift in composition was also observed by principal coordinate analysis of unweighted UniFrac distances measuring the overall diversity between samples, while the microbiota compositions remained relatively stable in the control group (Figures 1D,E and Supplementary Figures S1A,B,D,E). These observations were confirmed by pairwise comparisons of unweighted UniFrac distances between different days within the same group, which, however, also revealed a minor temporal effect in the control group (Figure 1F).

### Amoxicillin Affects Small Intestine and Cecal/Fecal Microbiota Compositions Differently

**FIGURE 2:**
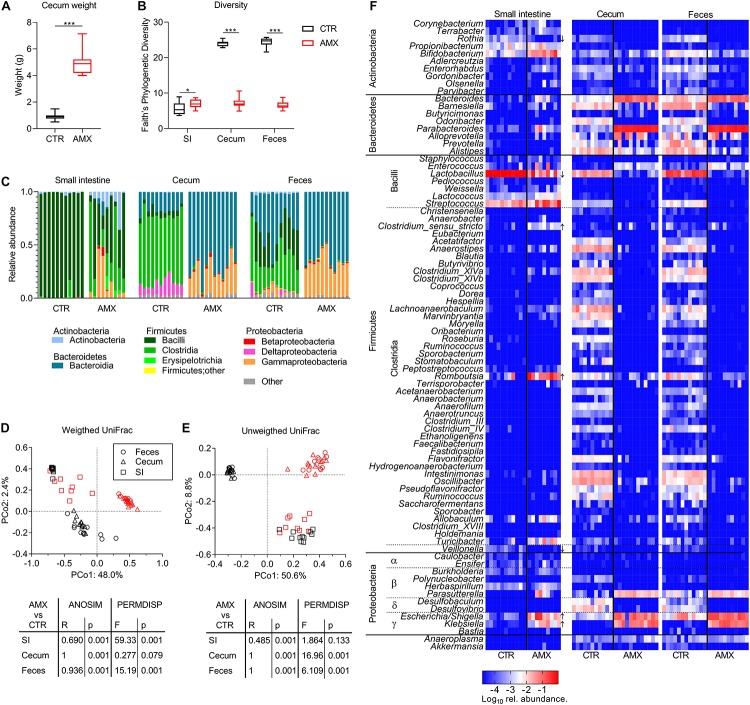
Effects of amoxicillin on intestinal microbiota. **(A,B)** Cecum weight **(A)** and Faith’s phylogenetic diversity **(B)** of control (CTR, black) and amoxicillin (AMX, red) groups. **(C)** Relative abundance of bacterial classes in individual animals. **(D,E)** Principal coordinate analysis (PCoA) plot of weighted **(D)** and unweighted **(E)** and UniFrac distances between samples from small intestine (SI) (squares), cecum (triangles), and fecal (circles) content day 7 colored according to CTR and AMX treatment. **(F)** Relative abundance of bacterial genera in individual animals indicated by the cell color ranging from highly abundant genera in red and less abundant genera in blue. Genera for which the abundance was significantly different between CTR and AMX groups in the small intestine are highlighted with arrows marking whether the genera were higher or lower after amoxicillin administration. Statistically significant differences between groups analyzed by non-parametric Mann–Whitney test **(A)** or *t*-tests **(B)** are indicated as *p ≤ 0.05, ***p ≤ 0.001.

After 7 days of amoxicillin administration, animals were euthanized and the effects on intestinal microbiota in different intestinal compartments were analyzed. Amoxicillin was found to have a profound physiological effect on the ceca, which were dramatically enlarged (Figure 2A), indicating changes in microbiota composition and activity. Indeed, similar to the fecal microbiota, the Faith’s phylogenetic diversity of the cecal microbiota was much lower in the amoxicillin group compared to the control group on day 7 (Figure 2B). In contrast, the small intestine diversity was higher in the amoxicillin group compared to the control group.

The distribution of bacterial classes was found to vary between the small intestinal, cecal and fecal compartments within the control group at day 7 (Figure 2C). The small intestine was dominated almost exclusively by Bacilli (lactobacilli), whereas three different classes dominated the cecal (Bacteroidia, Clostridia, and Deltaproteobacteria) and fecal (Bacteroidia, Clostridia, and Bacilli) microbiota. Amoxicillin administration was found to dramatically, and differently alter the microbiota in the different intestinal compartments. In the small intestine, the relative abundance of Bacilli was reduced while Gammaproteobacteria, Clostridia, and Actinobacteria were increased in all animals, but to a varying degree, resulting in a high variation between individual animals in the amoxicillin group. In contrast, the cecal and fecal microbiota composition were found to be uniform among the individual animals in the amoxicillin group, and the distribution of bacterial classes remarkably similar between the cecal and fecal microbiota in the amoxicillin group. In feces and cecum, the relative abundances of Clostridia and Bacilli were lower in the amoxicillin group than in the control group, while the relative abundance of Bacteroidia and Gammaproteobacteria were increased.

In line with the dramatic effect of amoxicillin on bacterial class distribution, the overall bacterial community structure (β diversity) was significantly affected by amoxicillin in both the small intestine, cecum and feces as determined by the ANOSIM method (Figures 2D,E). Furthermore, analysis of sample dispersion by PERMDISP of weighted UniFrac distances confirmed that the individual animals’ small intestinal samples were significantly more dispersed in the amoxicillin group than seen in the controls, while in contrast, cecal and fecal samples were significantly less dispersed in the amoxicillin group than in controls (Figure 2D). In summary, amoxicillin reduced within sample (α) and between samples (β) diversity with regard to the overall bacterial composition (weighted UniFrac distances) in fecal and cecal samples, but increased α and β diversity in small intestine samples.

Opposite, analysis of unweighted UniFrac distances based on presence/absence status of ASVs and not relative abundances revealed that the cecal and fecal samples from individual animals were significantly more dispersed in the amoxicillin group than in controls (Figure 2E). This suggests that while amoxicillin caused the amount of dominating species to become more similar within the group, the presence/absence status of rare species became more dissimilar within the group.

Statistical analysis of compositional changes (ANCOM method) of the microbiota caused by amoxicillin was performed only for the small intestinal compartment due to the very pronounced effects in the cecum and feces. The abundance of *Lactobacillus* was significantly lower in the amoxicillin group, as were *Veillonella* and *Rothia* (Figure 2F and Supplementary Table S1). A significant outgrowth of *Escherichia/Shigella* and *Klebsiella* (Gammaproteobacteria) in response to amoxicillin was also observed in the small intestine similar to the cecal and fecal compartments. Furthermore, the relative abundances of the Clostridial genera *Romboutsia* and *Clostridium sensu stricto* were significantly higher within the small intestine in the amoxicillin group than in the control group.

### Amoxicillin Promotes Host Mucosal Immune Regulation

**FIGURE 3:**
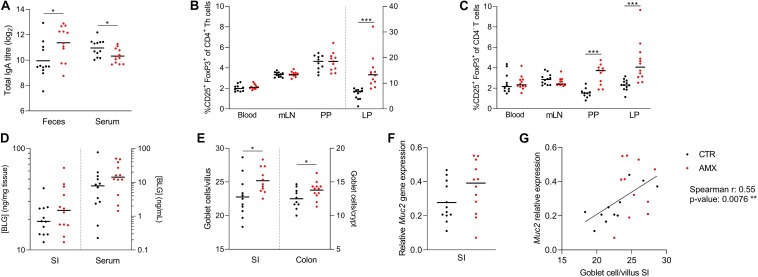
Host responses to amoxicillin. **(A)** Total IgA levels in feces and serum. **(B,C)** Relative amounts of CD25^+^ FoxP3^+^ T regulatory cells of CD4^+^ T helper cells **(B)** and CD4^–^ T cells **(C)** in blood, and small intestine mesenteric lymph nodes (mLN), Peyer’s patches (PPs) and lamina propria (LP). Cells in LP are plotted according to the right *y*-axis while cell from the remaining tissues are plotted according to the left *y*-axis. **(D)** β-lactoglobulin (BLG) concentration in small intestine LP (left *y*-axis) and serum (right *y*-axis). **(E)** Average goblet cells per villi or crypt in histological slides of small intestine (left *y*-axis) and colon (right *y*-axis). **(F)** Expression of the mucin 2 (*Muc2*) gene relative to two housekeeping genes. **(G)** Correlation between number small intestine goblet cells and relative expression of *Muc2*. Each symbol represents one animal and horizontal lines indicate mean **(A,E,F)** or median **(B–D)**. Statistically significant differences between control (CTR, black) and amoxicillin (AMX, red) groups analyzed by *t*-tests **(A,E)** or non-parametric Mann–Whitney test **(B,C)** are indicated as *p ≤ 0.05, ***p ≤ 0.001.

The effects of amoxicillin on humoral and mucosal immune regulation were analyzed after 7 days of administration. This revealed that total fecal IgA levels were higher in the amoxicillin group than in the control group, while total serum IgA levels were lower (Figure 3A). Various lymphocyte populations were analyzed in blood, mLNs, small intestine PPs and LP (Supplementary Figures S3A–C). Of these, the relative amount of CD25^+^FoxP3^+^ Tregs were significantly higher in small intestine LP (both CD4^+^ and CD4^–^ Tregs) and PPs (only CD4^–^ Tregs) in the amoxicillin group compared to the control group, while amoxicillin had no effect on Tregs in blood and mLNs (Figures 3B,C). Small intestinal uptake of the cow’s milk protein BLG was assessed by quantifying BLG in gut tissue and serum 15 min after oral gavage with whey protein. This revealed a tendency for higher uptake in the amoxicillin group (*p* = 0.183 and *p* = 0.139 for LP and serum, respectively; Figure 3D). Finally, mucus-producing goblet cells were quantified in histological slides (representative pictures in Supplementary Figure S4). Goblet cell numbers in both the small intestine and colon were elevated in the amoxicillin group compared to controls (Figure 3E). Relative expression of various immune related host genes were assessed in small intestine LP (Supplementary Figure S3D), and a tendency (*p* = 0.131) for higher expression of the *Muc2* gene encoding mucin 2 was observed in the amoxicillin group (Figure 3F). A strong positive correlation between small intestine goblet cells and *Muc2* gene expression supported these findings (Figure 3G).

### Specific Bacterial Genera Are Linked to Immune Regulatory Responses

**FIGURE 4:**
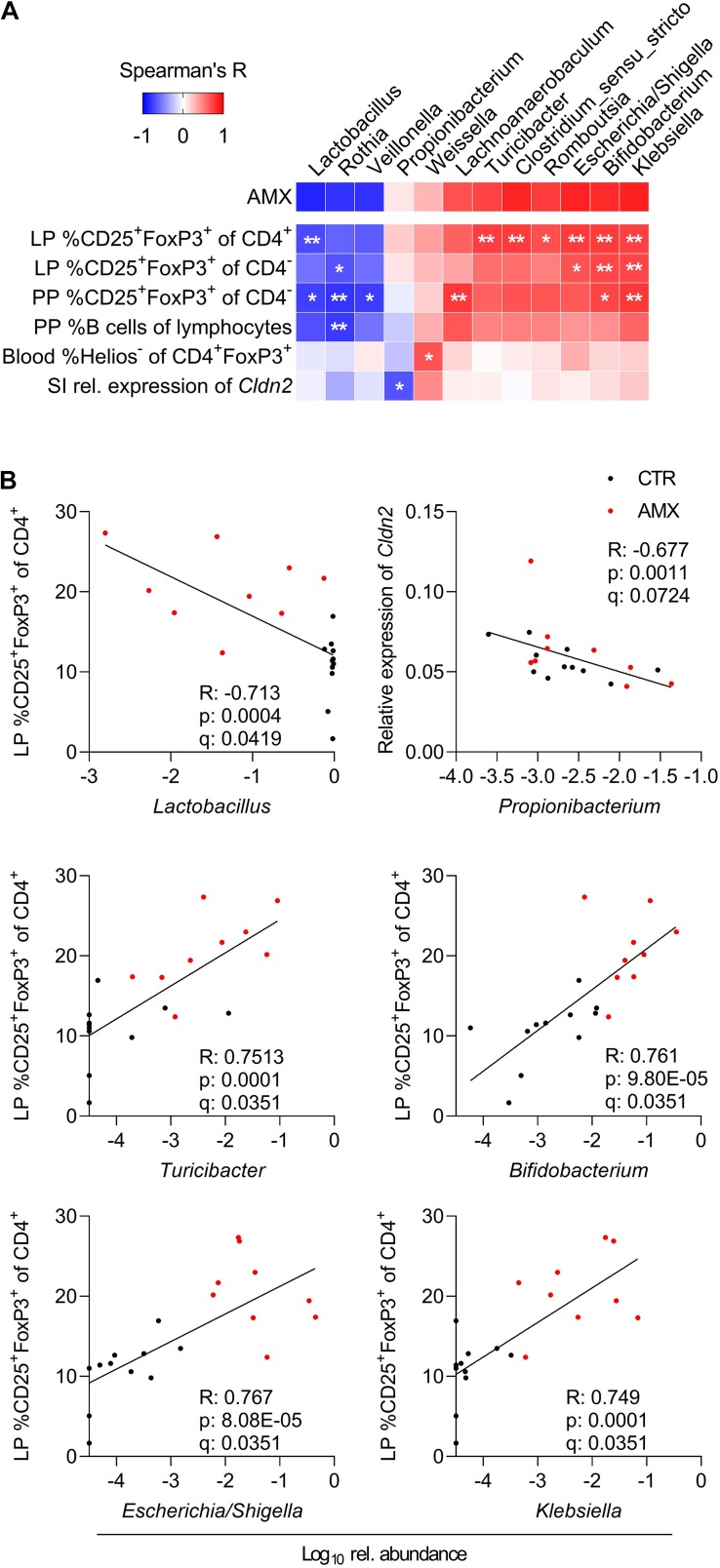
Correlations between small intestine bacterial genera and host immune responses. **(A)** Spearman’s Rank correlation matrix between relative abundance of small intestinal bacterial genera present in minimum 10 animals, and all host response parameters indicated on Figure 3 and Supplementary Figure S3 including small intestinal (SI) gene expression and lymphocyte populations in lamina propria (LP), Peyer’s patches (PP) and blood. Only those parameters for which correlations are significant after applying FDR-correction are included. Spearman’s Rank correlation coefficients are indicated by the cell color. Statistically significant differences are indicated as *q < 0.1, **q < 0.05. **(B)** Visualization of selected correlations between relative abundance of small intestine genera and host responses in control (CTR, black) and amoxicillin (AMX, red) administered group. Each symbol represents one animal.

To investigate the interplay between the bacterial composition of the small intestine and the host immune response, pairwise correlations between relative abundance of genera and the host immune parameters shown in Figure 3 and Supplementary Figure S3 were performed (Figures 4A,B). This revealed negative correlations between three bacterial genera with reduced relative abundance in the amoxicillin group, namely *Lactobacillus*, *Rothia*, and *Veillonella*, and the fraction of both CD25^+^FoxP3^+^ Tregs in the small intestine LP (both CD4^+^ and CD4^–^ Tregs) and PPs (only CD4^–^ Tregs) as well as with the fraction of B cells of all PPs lymphocytes. Further, a consortium of *Escherichia/Shigella*, *Klebsiella*, *Bifidobacterium*, and four Firmicutes genera for which the relative abundance was higher in the amoxicillin group compared to controls were positively correlated with the same lymphocyte populations. These observations were supported by the additional finding of a significant correlation between LP CD4^+^ Tregs and *Escherichia/Shigella* in the control group only (*p* = 0.035) and for *Turicibacter* in the amoxicillin group only (*p* = 0.014). In addition, a negative correlation was found between the relative abundance of *Propionibacterium* and the expression of the *Cldn2* gene encoding the tight junction protein Claudin 2 in the small intestine tissue, and a positive correlation between the relative abundance of *Weissella* and the fraction of Helios^–^ induced Tregs in circulation.

## Discussion

To investigate the putative relationship between microbiota composition and immune regulation, BN rats were orally administered with the antibiotic amoxicillin or water as control for 7 days, and the gut microbiota, host immune regulation and intestinal permeability to cow’s milk protein were assed. Despite of epidemiologic studies showing an association between early life antibiotic consumption and prevalence of food allergies (Metsälä et al., 2013; Hirsch et al., 2017; Mitre et al., 2018) and autoimmune disorders such as IBD (Hviid et al., 2011; Shaw et al., 2011; Kronman et al., 2012), reflecting a failure to develop appropriate tolerance, the current study surprisingly indicated that a 7 days amoxicillin-induced perturbation of the gut microbiota promoted multiple parameters associated with immune regulation. These included higher relative abundance of small intestine Tregs, total fecal IgA and goblet cell numbers in the amoxicillin group compared to controls.

The observed increase in goblet cell numbers is in agreement with a previous study showing that administration the board-spectrum antibiotic ceftriaxone to Wistar rats causes increased goblet cell numbers and size (Holota et al., 2019). It is well established that bacterial colonization affects mucin gene expression, and in agreement with the present study, a negative correlation between abundance of *Lactobacillus* spp. and *Muc2* gene expression in newborn mice has previously been reported (Bergström et al., 2012). Goblet cells promote oral tolerance by delivering luminal antigens to tolerogenic CD103^+^ dendritic cells in the small intestine to generate antigen specific Tregs (McDole et al., 2012; Shan et al., 2013) and by producing mucus, which contributes to intestinal barrier function and provides attachment sites and nutrients for commensal gut bacteria (Martens et al., 2009).

In the current study, the relative abundance of CD25^+^FoxP3^+^ Tregs in the small intestine LP (both CD4^–^ and CD4^+^) and PPs (only CD4^–^ Tregs) were found to be higher in the amoxicillin group than in controls. While conventional CD4^+^ Tregs have been extensively studied, the function of CD8^+^CD4^–^ Tregs remains less characterized, but are thought to play an important role in preventing food allergy (Yamada et al., 2009). In line with the results from the current study, oral administration of broad-spectrum antibiotic cocktails is often reported to increase the relative abundance of CD4^+^ Treg cells in small intestine (Ivanov et al., 2008; Ochoa-Repáraz et al., 2009; Ichinohe et al., 2011; Janelsins et al., 2014; Benakis et al., 2016; Nakamura et al., 2016; Strzępa et al., 2017), and to reduce relative abundance of Th1 (Ochoa-Repáraz et al., 2009; Hill et al., 2010) and/or Th17 effector cells (Ivanov et al., 2008; Ochoa-Repáraz et al., 2009; Hill et al., 2010; Janelsins et al., 2014; Benakis et al., 2016). This shift in the balance between Tregs and effector T cells has been shown to reduce severity of multiple autoimmune (Ochoa-Repáraz et al., 2009; Benakis et al., 2016; Nakamura et al., 2016) and allergic diseases (Strzępa et al., 2017). On the other hand, the antibiotic-induced Treg promotion has been shown to prevent the development of proper immune responses to oral vaccination (Hall et al., 2008) and enteric infections (Sekirov and Finlay, 2009; Thackray et al., 2018).

Tregs are central stimulators of mucosal IgA production by plasma cells (Feng et al., 2011), which is reflected in the current study, since the higher relative abundance of small intestinal Tregs in the amoxicillin group than in controls, was accompanied by higher fecal IgA levels. Mucosal IgA is known to be important for the immunological defense against pathogenic bacteria and toxins, while the role of IgA in immune tolerance is less understood (Tordesillas and Berin, 2018). Still, results from human (Kukkonen et al., 2010) and animal (Frossard et al., 2004) studies indicate that IgA might play an important role for tolerance development, and alterations in IgA coating patterns of intestinal bacteria have been associated with multiple disorders including atopic diseases (Dzidic et al., 2017) and IBD (Van Der Waaij et al., 2004), although the results are ambiguous. Opposite to what was observed for fecal IgA, total serum IgA levels were significantly lower in the amoxicillin group than in controls. This is in line with previous studies showing that fecal and serum IgA levels are not affected in the same way by oral exposure to allergens (Frossard et al., 2004) and high-fat diet (Luck et al., 2019) in mice. Amoxicillin-induced perturbation of the microbiota may affect the transport of IgA from circulation to the intestinal lumen and/or tissue specific differences in regulation of IgA secretion.

To investigate the interplay between microbiota composition and immune regulation, the fecal microbiota was analyzed throughout the study, and the small intestinal and cecal microbiota at termination. Amoxicillin was found to dramatically, and differently alter the microbiota in the different intestinal compartments. The small intestine was vastly dominated by *Lactobacillus* in the control group thus this compartment had lower α diversity compared to feces and cecum. This genus was much less abundant in the amoxicillin group, where multiple different genera were more abundant with high variation between individual animals causing a higher α and β diversity compared to controls. This was in contrast to the fecal and cecal microbiota, for which both α and β diversity were significantly lower in the amoxicillin group compared to controls. These differences in response probably result from differences in bacterial load, diversity and composition in the different compartments (Hayashi et al., 2005; Wang et al., 2005; Seekatz et al., 2019). Differences in the transit time, which is much faster in the small intestine compared to cecum and colon (Wang et al., 2015), imply a shorter contact time between bacteria and antibiotics, which may also have affected the response. The current study highlights that changes in fecal bacterial population do not provide a good proxy for small intestinal responses to antibiotics.

Common for all analyzed intestinal compartments was the reduction in relative abundance of *Lactobacillus* and increase in relative abundance of Gammaproteobacteria. This was also seen in feces after 7 days treatment of healthy adults with amoxicillin in combination with β-lactamase inhibitor clavulanate (Kabbani et al., 2017; MacPherson et al., 2018). The elevated level of facultative anaerobes within the Gammaproteobacteria may in part be explained by depletion of butyrate producing clostridia, since reduction in butyrate has been reported to promote expansion of oxygen tolerating species due to increased epithelial oxygenation (Rivera-Chávez et al., 2016). In line with this explanation, previous studies report reduced butyrate levels and elevated cecal pH after amoxicillin administration in Wistar rats (Tulstrup et al., 2015, 2018).

**FIGURE 5:**
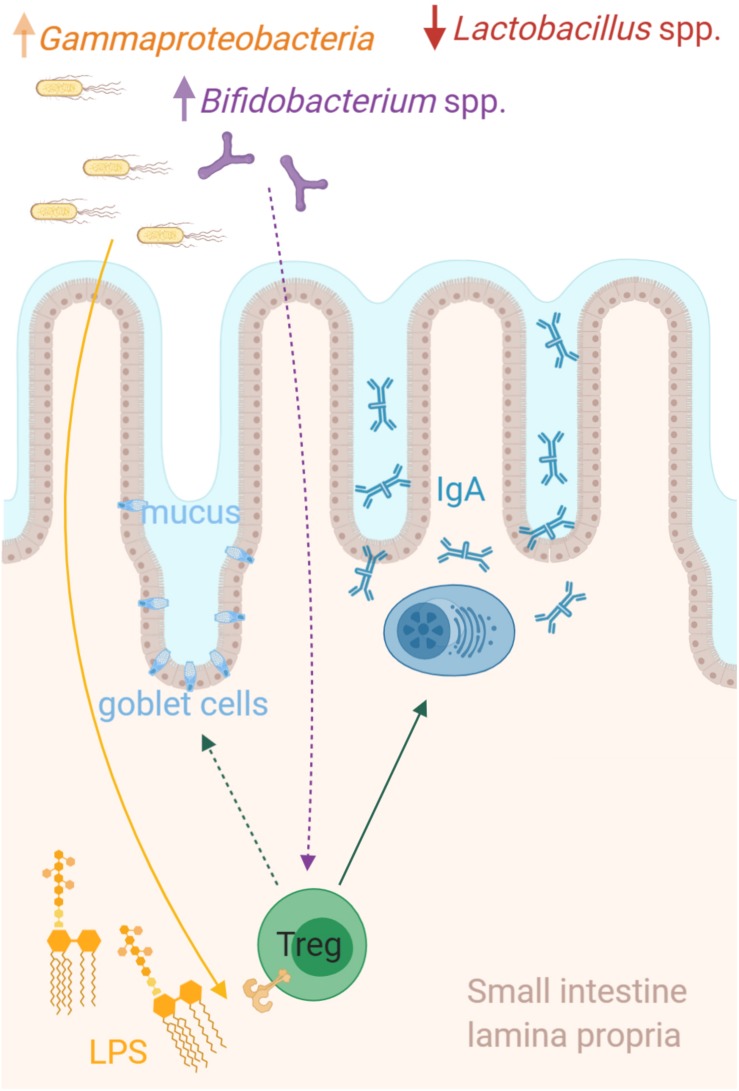
Microbiota host interactions in the small intestine. The relative abundance of *Lactobacillus* was dramatically lower in the small intestine of amoxicillin administered rats, while multiple different genera were more abundant. Among these were a consortium including *Escherichia/Shigella*, *Klebsiella* (Gammaproteobacteria), and *Bifidobacterium*, which strongly correlated with the relative abundance of regulatory T cells (Tregs) in the small intestine. We speculate that bacterial derived signals such as hexa-acylated lipopolysaccharide (LPS) from Gammaproteobacteria, could directly promote the expansion of the intestinal Tregs, which in turn stimulate mucosal IgA production by local plasma cells and possibly goblet cell activation and mucus secretion. The figure is created with BioRender.com.

Multiple studies have revealed that bacterial stimulation is important for mucosal T cell responses and in particular important for balancing the ratio between Tregs and effector T (Lee and Kim, 2017). In the current study, a consortium consisting of *Escherichia/Shigella*, *Klebsiella* (Gammaproteobacteria), *Bifidobacterium*, and four genera belonging to Firmicutes, for which the relative abundance in the small intestine were higher in the amoxicillin group than in controls, positively correlated with the fraction of small intestinal Tregs. This is in accordance with previous studies reporting positive effects of administration with various probiotic *Bifidobacterium* spp. on the Treg/T effector cell balance in humans (Konieczna et al., 2012) and mice, which thereby ameliorate allergic and autoimmune diseases (Lyons et al., 2010; Zuo et al., 2014; Liu et al., 2018; Kim et al., 2019). Furthermore, a growing body of evidence suggests an important immunomodulatory role of hexa-acylated lipopolysaccharide (LPS), a major constituent of the outer membrane of Gammaproteobacteria, which exhibit superior Toll-like receptor 4 (TLR4) stimulatory capacity compared to the less acylated LPS present from other Gram-negative bacteria (Larsen et al., 2012, 2015; Li et al., 2013; Brix et al., 2015). We speculate that bacterial derived signals such as hexa-acylated LPS from Gammaproteobacteria could directly promote the expansion of the intestinal Tregs, which in turn stimulate mucosal IgA production by local plasma cells (Figure 5). Indeed, *in vitro* studies have shown that LPS can activate murine (Caramalho et al., 2011) and human (Lewkowicz et al., 2016) Tregs directly via TLR4 activation. Furthermore, animal studies have indicated a link between high dietary LPS levels and higher numbers of CD4^+^ Tregs in mLN, as well as higher CD8^+^ Tregs in mLN and PPs (Hrncir et al., 2008). However, further studies are need to address this hypothesis, such as applying the same experimental treatment to LPS-non-responsive animals, to see if Treg and IgA induction fail to appear. One LPS-non-responsive animal model is TLR4^–/–^B6129PF mice, which are in fact prone to develop food allergy (Bashir et al., 2004).

## Conclusion

This study showed that microbiota perturbation by amoxicillin promotes acute intestinal immune regulation. The observed immune regulation may represent an acute response to overt inflammatory signals derived from the microbiota, e.g., released from dead bacteria and/or new or expanded bacterial species. The long-term immunological and clinical consequences of this effect remain to be investigated to determine whether this condition affects development of oral tolerance to luminal antigens and thus prevents inflammatory and allergic diseases.

## Data Availability Statement

The 16S rRNA gene sequence data are deposited in the NCBI Sequence Read Archive with the accession number PRJNA599292.

## Ethics Statement

Ethical approval was given by the Danish Animal Experiments Inspectorate and the authorization number given 2015-15-0201-00553-C1. The experiment was overseen by the National Food Institute’s in-house Animal Welfare Committee for animal care and use.

## Author CONTRIBUTIONS

KB and TL conceived of the study. KG analyzed serum antibody levels, protein uptake, and histology. MB and KG analyzed microbiota data. JL analyzed the lymphocyte populations and participated in interpretation of the results. A-SB analyzed the gene expression. KG, MB, TL, and KB participated in the design of the study and the interpretation of the results. KG drafted the manuscript. All authors made substantial intellectual contributions, revised the manuscript critically, and approved the final version of the manuscript.

## Conflict of Interest

The authors declare that the research was conducted in the absence of any commercial or financial relationships that could be construed as a potential conflict of interest.
