# Supplementary material for: Short-Term Amoxicillin-Induced Perturbation of the Gut Microbiota Promotes Acute Intestinal Immune Regulation in Brown Norway Rats
Source: Front Microbiol. 2020 Mar 26;11:496. doi: 10.3389/fmicb.2020.00496 (PMC7135894; doi:10.3389/fmicb.2020.00496)
Supplement: Supplementary file 1 [file Data_Sheet_1.PDF]

Supplementary figures

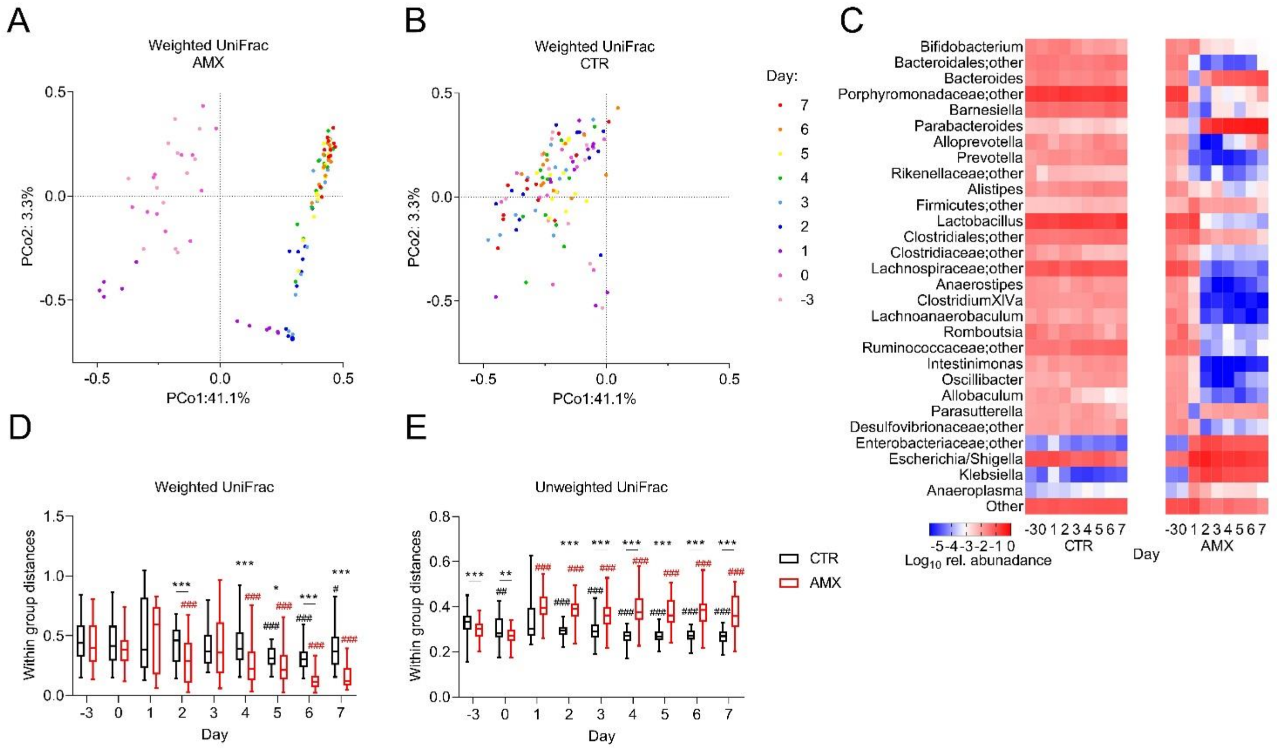

Fig. S1: A-B: Principal coordinate analysis (PCoA) plots of weighted UniFrac distances in control (CTR, A) and amoxicillin (AMX) group (B) colored according to sample day. C: Heat-map colored according to mean relative abundance of bacterial genera at different days. D-E: Within group distances based on weighted (D) and unweighted (E) UniFrac distances.

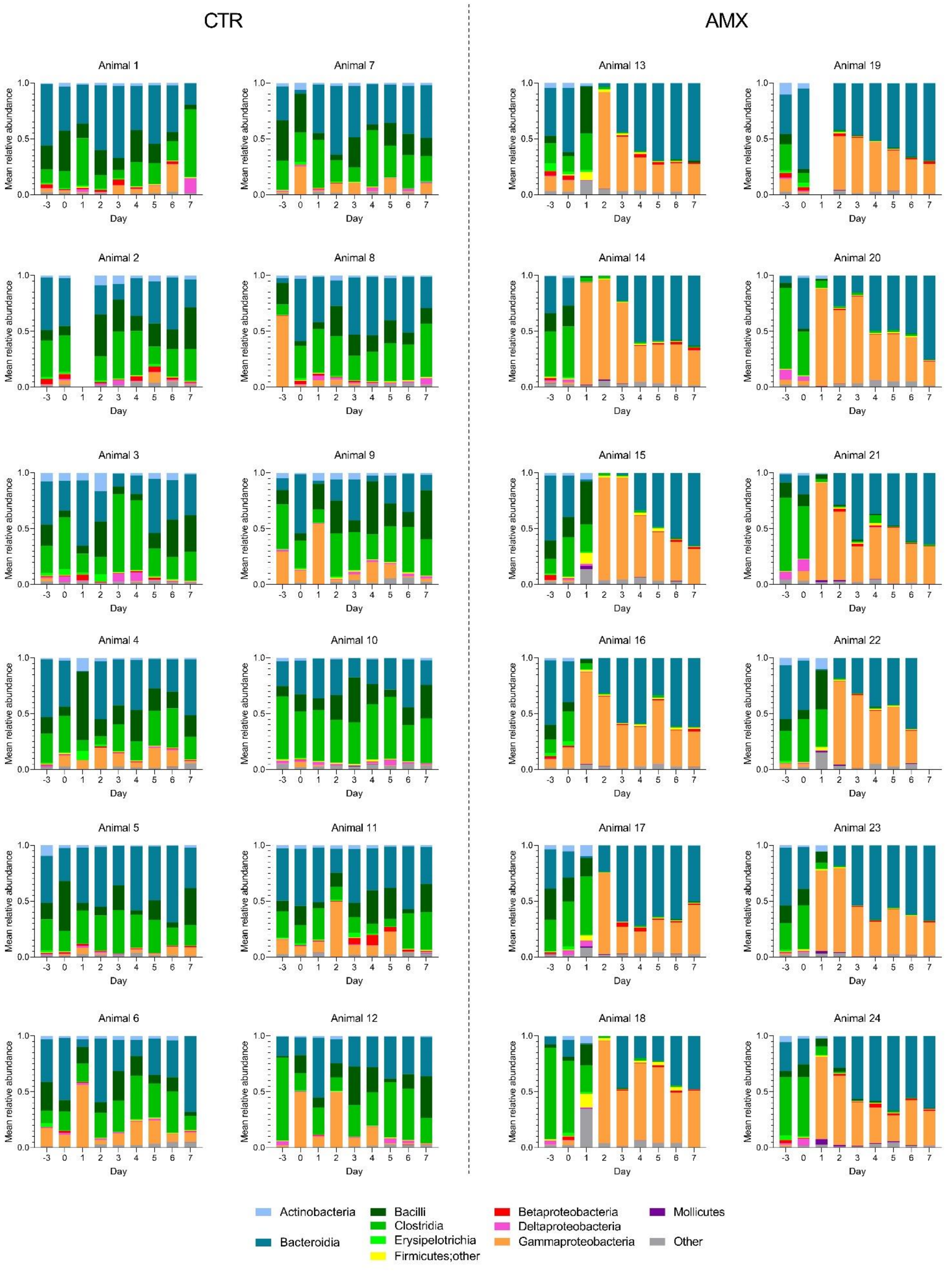
CTR
AMX
Animal 1
Animal 7
Animal 13
Animal 19
Animal 2
Animal 8
Animal 14
Animal 20
Animal 3
Animal 9
Animal 15
Animal 21
Animal 4
Animal 10
Animal 16
Animal 22
Animal 5
Animal 11
Animal 17
Animal 23
Animal 6
Animal 12
Animal 18
Animal 24
Mean relative abundance
Day
Actinobacteria
Bacteroidia
Bacilli
Clostridia
Erysipelotrichia
Firmicutes;other
Betaproteobacteria
Deltaproteobacteria
Gammaproteobacteria
Mollicutes
Other

Fig. S2: Temporal effects of amoxicillin on relative abundance of bacterial classes in individual animals.

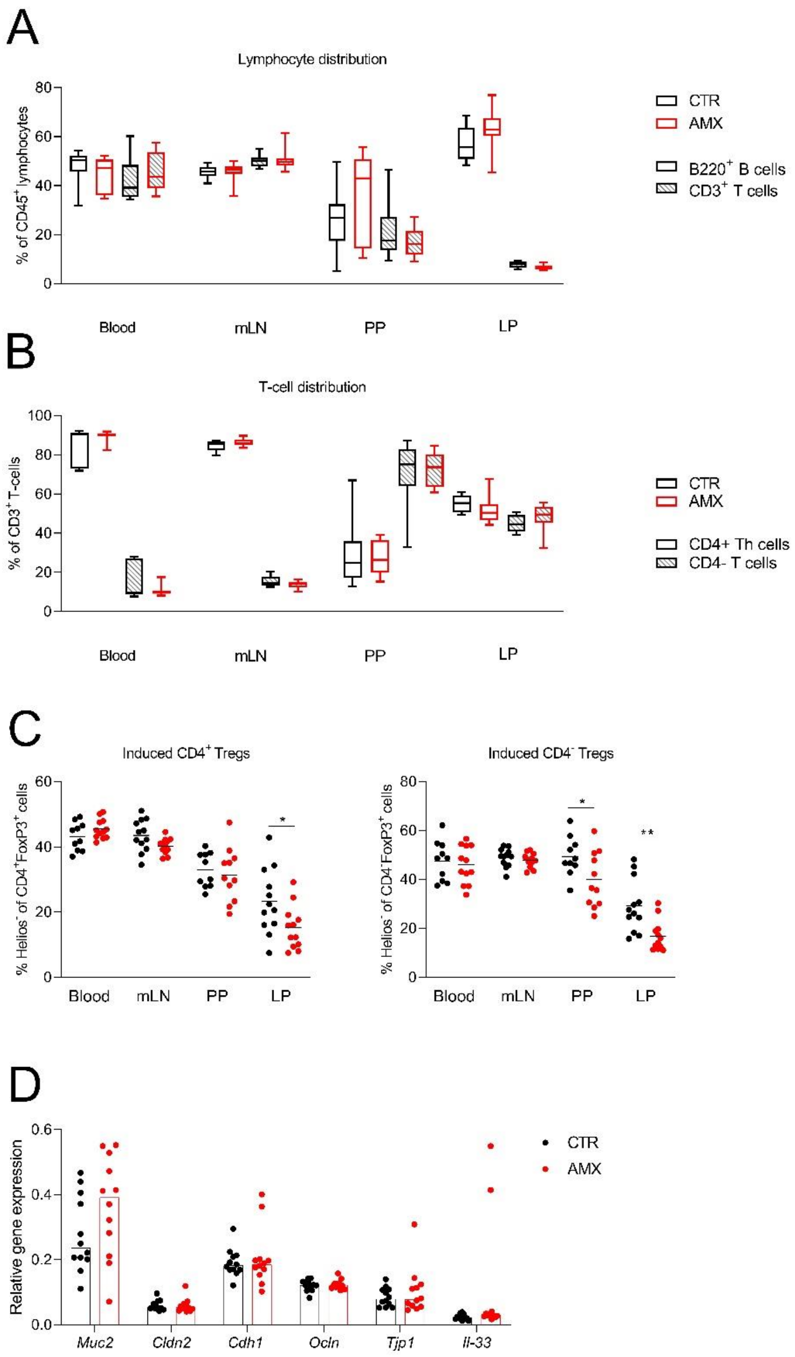

Fig. S3: Relative distribution of lymphocyte populations in blood, mesenteric lymph nodes (mLN), lamina propria (LP) and Peyer's patches (PP) (A) and relative gene expression in small intestine LP of control (red) and amoxicillin administered (red) animals. A: Percentage B220^{+} B cells (empty bars) and CD3^{+} T cells (hatched bars) of all lymphocytes. B: Percentage CD4^{+} T helper cells (empty bars) and

CD4$^-$ T cells (hatched bars) of all CD3$^+$ T cells. C: Percentage Helios$^-$ cells of all CD4$^+$ and CD4$^-$ FoxP3$^+$ T regulatory (Treg) cells. D: Gene expression of mucin 2 (*Muc2*), claudin 2 (*Cldn2*), cadherin 1 (*Cdh1*), occluding (*Ocln*), zonula occludens/tight junction protein 1 (*Tjp1*) and interleukin 33 (*Il33*) relative to housekeeping genes.

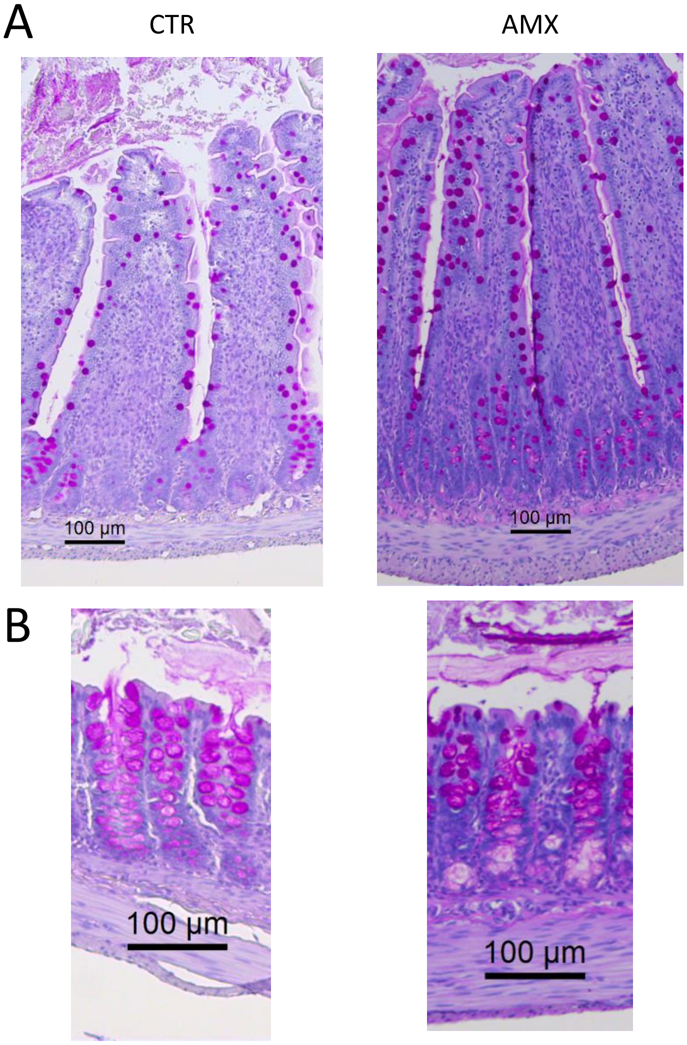

Fig. S4: Representable pictures of goblet cell staining by Periodic acid–Schiff (PAS) in small intestinal (A) and colon (B) sections.

Table S1: Differentially abundant genera between small intestine of control and amoxicillin administered rats analyzed by analysis of composition of microbiomes (ANCOM).

	Genera	**W**	**clr**
**Statistically significant**	*Lactobacillus*	81	-3.9796866193279854
	Escherichia/Shigella	78	4.3408022010976515
	Rothia	78	-2.673502453407982
	Romboutsia	74	4.830549788692652
	Klebsiella	69	3.236854263583691
	Veillonella	63	-1.967662858164571
	Clostridium_sensu_stricto	63	2.09302398153761
**Borderline statistically significant**	*Bifidobacterium*	55	2.4258928100122437
